# Superficial femoral artery–anterior tibial artery bypass with great saphenous vein grafting via the lateral femoropopliteal route for infection after Viabahn placement

**DOI:** 10.1002/ccr3.7629

**Published:** 2023-06-28

**Authors:** Saeki Watanabe, Hironobu Morimoto, Daisuke Futagami, Junya Kitaura, Syogo Mukai, Taira Kobayashi

**Affiliations:** ^1^ Department of Cardiovascular Surgery Fukuyama Cardiovascular Hospital Fukuyama‐shi, Hiroshima Japan; ^2^ Department of Cardiovascular Surgery JA Hiroshima General Hospital Hatsukaichi, Hiroshima Japan

**Keywords:** aneurysm, peripheral arterial disease, vascular surgical procedures

## Abstract

We report a case involving an elderly man who successfully underwent superficial femoral artery–anterior tibial artery bypass via the lateral femoropopliteal route following development of a stent infection after placement of a small‐diameter covered stent for a ruptured superficial femoral artery pseudoaneurysm. This report suggests that appropriate treatment strategies for device infection subsequent to device removal are paramount for the prevention of reinfection and preservation of the affected extremity.

## 1 INTRODUCTION

Device infection in the peripheral vascular area is rare, but it is often serious when it develops. Although device removal is necessary for treatment, methods to prevent reinfection while preserving the affected extremity must be considered for subsequent revascularization. Here, we report a case involving a 78‐year‐old man who developed a stent infection after placement of a small‐diameter covered stent for a ruptured superficial femoral artery pseudoaneurysm. The device had to be removed, and it was determined that revascularization was necessary to preserve the affected limb, along with bypass surgery. A lateral bypass route was chosen to keep the bypass graft away from the infected site. Thus, the patient was successfully treated with superficial femoral artery–anterior tibial artery bypass via the lateral femoropopliteal route.

## 2 CASE REPORT

A 78‐year‐old man with a history of Type 2 diabetes mellitus presented with a chief complaint of left thigh pain at a hospital and was subsequently transferred to our hospital for further diagnosis and treatment. The patient provided written informed consent for publication of his case and associated images.

**FIGURE 1:**
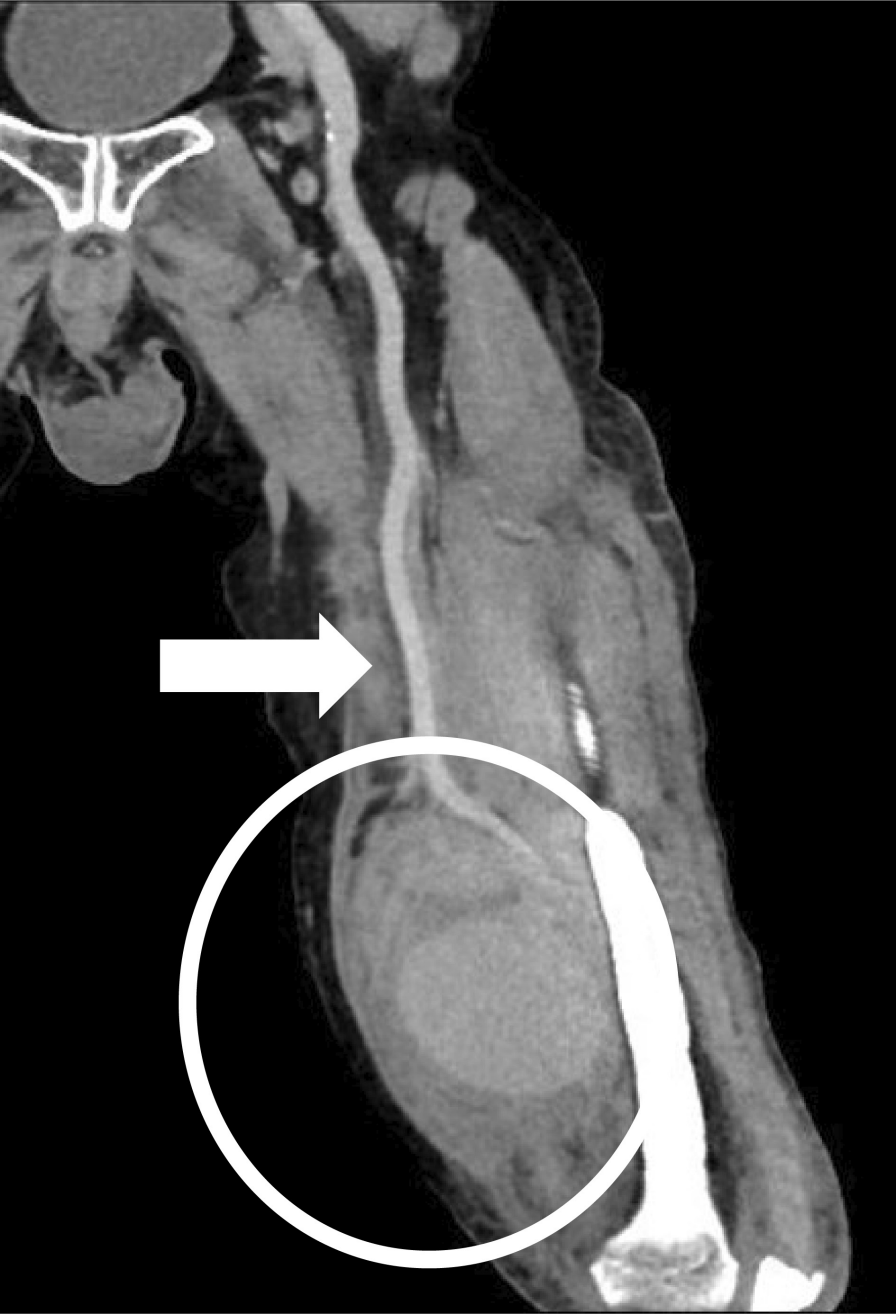
Preoperative contrast‐enhanced CT image of a 78‐year‐old man with superficial femoral artery pseudoaneurysm. The white arrow points to the left common femoral artery. Leakage of contrast medium and a hematoma can be seen (white circle).

**FIGURE 2:**
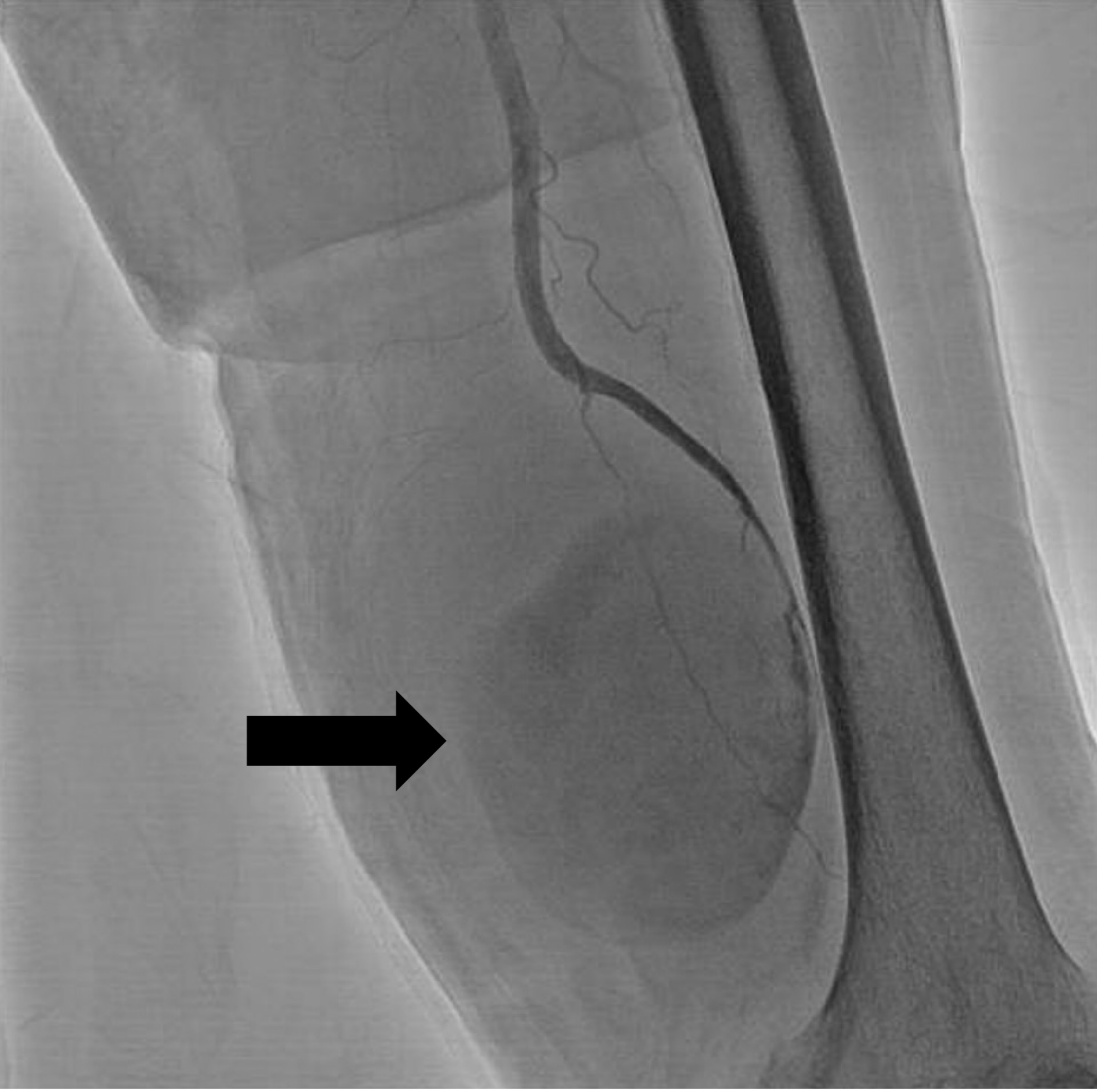
Angiography findings for a 78‐year‐old man with superficial femoral artery pseudoaneurysm. Leakage of contrast medium from the superficial femoral artery can be observed.

**FIGURE 3:**
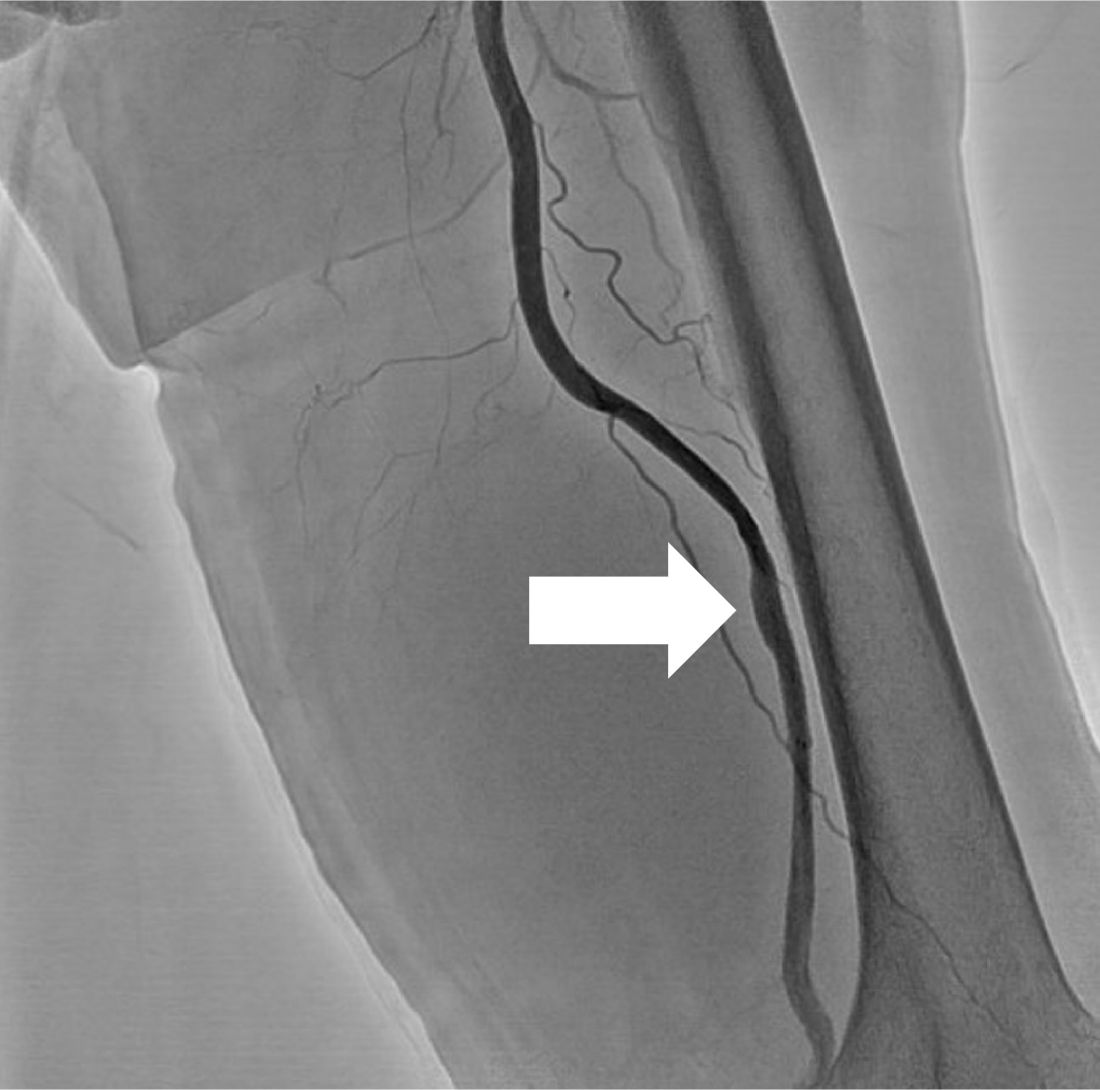
Angiography findings after Viabahn stent placement for a 78‐year‐old man with superficial femoral artery pseudoaneurysm. The white arrow shows findings suggesting disruption of the vessel wall structure.

**FIGURE 4:**
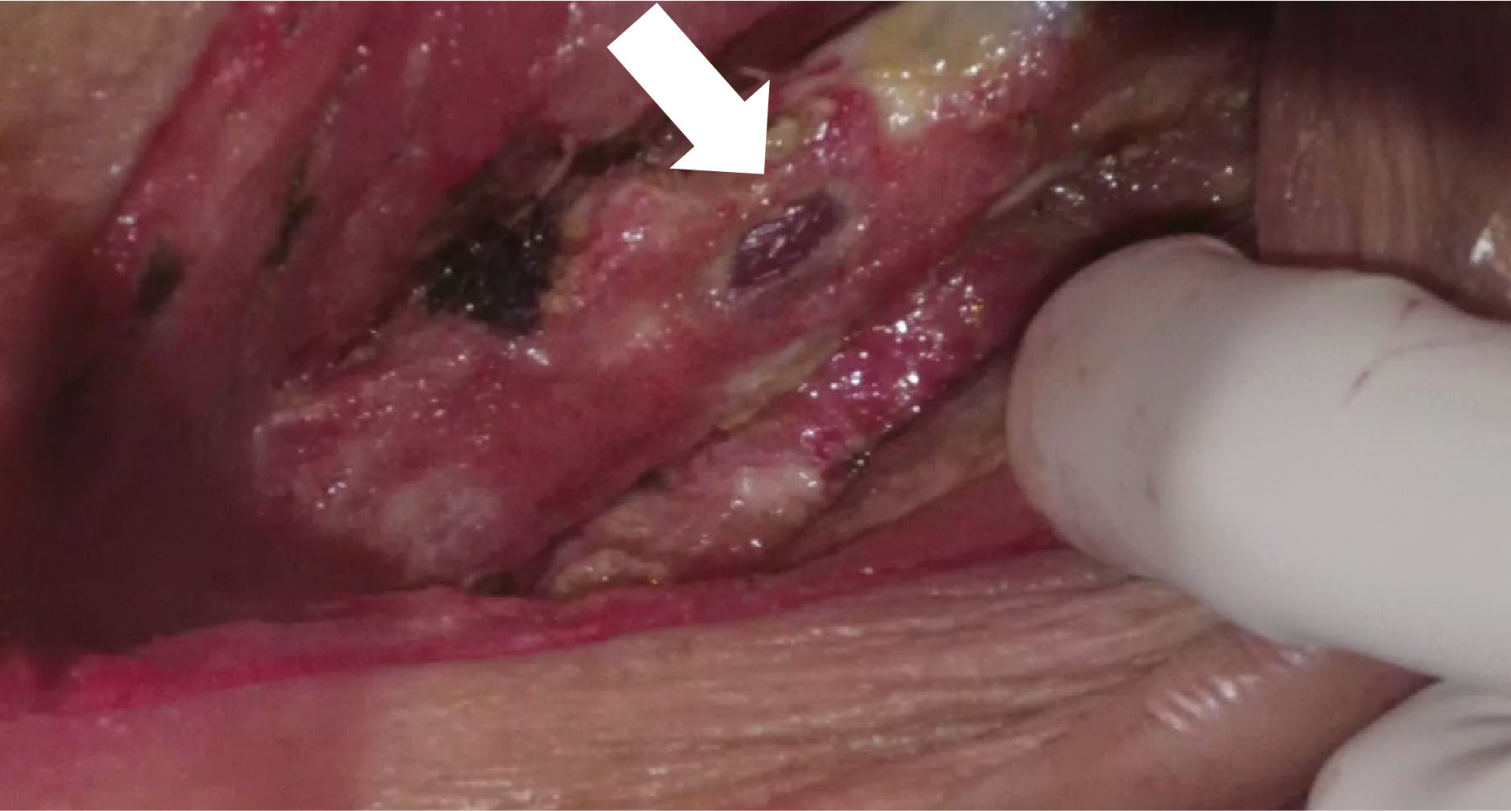
Intraoperative photograph for a 78‐year‐old man undergoing superficial femoral artery–anterior tibial artery bypass with great saphenous grafting via the lateral femoropopliteal route for Viabahn stent infection. The vessel wall is collapsed, and the stented portion of Viabahn is exposed.

**FIGURE 5:**
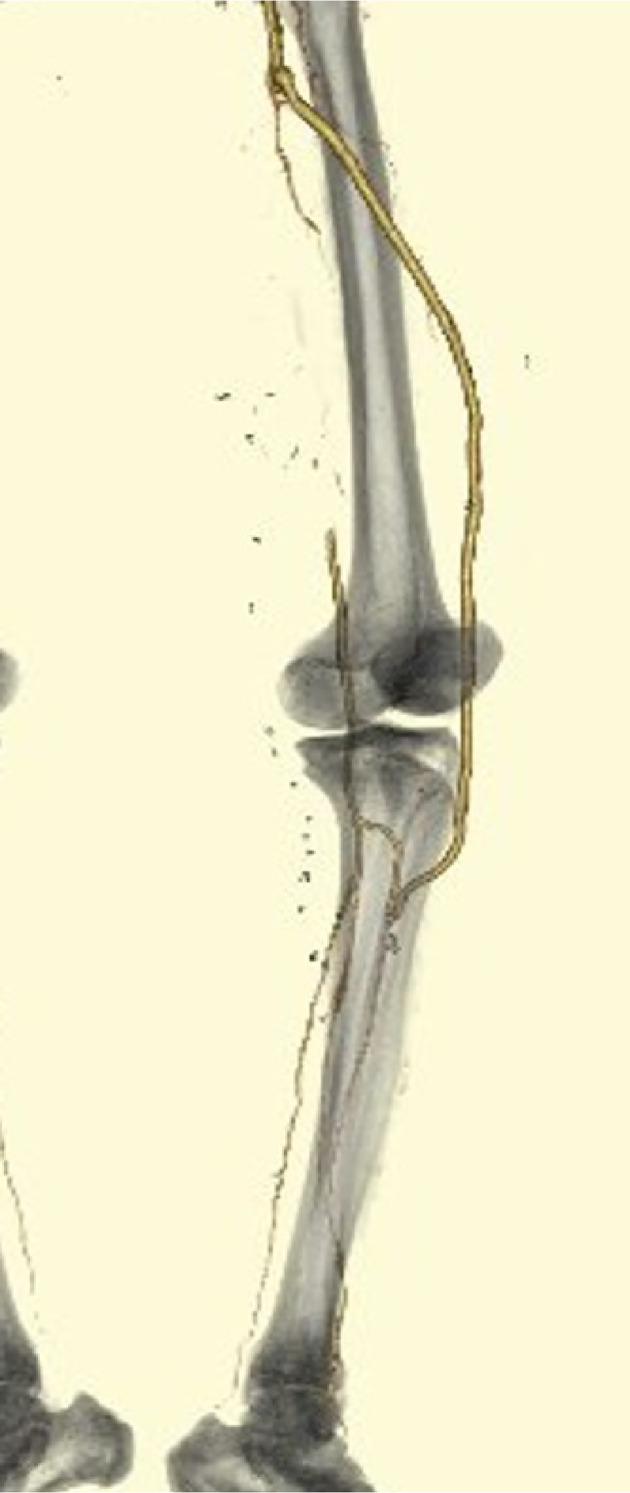
Postoperative contrast‐enhanced computed tomography image of a 78‐year‐old man who underwent superficial femoral artery–anterior tibial artery bypass with great saphenous grafting via the lateral femoropopliteal route for Viabahn stent infection. The image shows disappearance of the pseudoaneurysm, graft patency, and good contrast beyond the periphery.

On admission, the patient's body temperature was 37.7°C, with edema, erythema, and pain in the left thigh region. Blood test findings showed an increased white blood cell (WBC) count of 18,200/μL and a high C‐reactive protein (CRP) level of 21.09 mg/dL. Contrast‐enhanced computed tomography (CT) showed an intramuscular hematoma with a 10‐cm diameter in the left thigh. The popliteal artery was excluded by the hematoma from the distal part of the left superficial femoral artery, and the peripheral contrast effect was poor (Figure 1). Angiography showed rupture of the superficial femoral artery wall and leakage of contrast medium (Figure 2). Intravascular ultrasonography also showed a defect in the vessel wall, and we established a diagnosis of ruptured superficial femoral artery pseudoaneurysm. The area beyond the superficial femoral artery was occluded because of exclusion of the hematoma. Endovascular treatment was performed, and a Viabahn stent measuring φ6 mm × 50 mm (W.L. Gore and Associates) was placed from the superficial femoral artery to the popliteal artery. After stent placement, contrast medium leakage from the superficial femoral artery disappeared, and the contrast effect beyond the peripheral side improved. However, some findings suggesting collapse of the vascular wall structure were observed (Figure 3). After endovascular treatment, puncture drainage was performed for the hematoma in the left thigh. Sanguineous drainage was observed, and a drain was placed. Infection was suspected because of an increase in the inflammatory response, and follow‐up CT showed a residual hematoma in the left thigh with surrounding gas, which appeared to be produced by bacteria. Considering the active infection, we decided to remove the hematoma and perform lavage and drainage. When the inside of the left thigh was incised, a large amount of cream‐colored pus flowed out. Following abscess removal, we observed that the vascular wall of the superficial femoral artery had collapsed, and we also observed exposure of the stented portion of Viabahn (Figure 4). Accordingly, we decided to remove the infected stent and perform lower limb bypass surgery with grafting using the left great saphenous vein. A subcutaneous tunnel was created in the lateral femoropopliteal route to maintain as safe a distance from the infected site as possible. Next, after confirmation of vein dilatation, the graft was passed through the tunnel, the valve was destroyed using a valve cutter, and non‐reversed saphenous vein bypass grafting was performed. Graft patency was confirmed through angiography, and all wounds were closed. Subsequently, the infected Viabahn stent was removed, and negative pressure wound therapy was applied. Methicillin‐sensitive *Staphylococcus aureus* was detected in the intraoperative culture sample, and antibiotics were administered for 4 weeks. Postoperative contrast‐enhanced CT showed disappearance of the pseudoaneurysm with graft patency and good contrast beyond the periphery (Figure 5). The patient was discharged from the hospital 50 days after the surgery.

## 3 DISCUSSION

Causes of pseudoaneurysm include infection, trauma, iatrogenic causes, Behcet's disease, Ehlers–Danlos syndrome (Type IV), and other connective tissue disorders.^1^ Spontaneous onset of superficial femoral artery pseudoaneurysm is rare^2^ and thought to be caused by atherosclerosis.^3^ In this case, there was no evidence of atherosclerosis in the superficial femoral artery, which was in good condition. The cause of the superficial femoral artery pseudoaneurysm suspected to be infection; however, blood culture and aspiration effluent culture at the time of admission showed negative results, and there was no history of trauma or connective tissue disorders; this suggested the possibility of a spontaneous superficial femoral artery pseudoaneurysm. In the superficial femoral artery region, treatment with stent grafts may be effective, and Viabahn is considered an effective device for treatment.^4^ There have been some reports of spontaneous superficial femoral artery pseudoaneurysms treated with Viabahn,^2^, ^5^ similar to the present case.

Infections in Viabahn are reportedly rare.^6^ Fabrizio et al. reported a case involving Viabahn infection in which femoral artery–popliteal artery bypass was performed after infected device removal.^7^ Stent graft infection in the peripheral arterial region is rare; however, it can be serious when it develops, leading to conditions necessitating amputation of the affected limb or even death.^8^ Therefore, removal of the infected device is necessary. In the present case, several risk factors for infection were present, including diabetes, use of arterial puncture, hematoma drainage, and catheter placement. However, the inflammatory response was already exacerbated at the time of the patient's visit, and it was difficult to identify the cause of the infection. Intraoperative findings revealed rupture of the superficial femoral artery wall and Viabahn exposure. It is possible that the structure of the vessel wall was not maintained, and that continuity had been created outside the blood vessels, which facilitated infection. Muhip et al. recommend percutaneous drainage in endovascular treatment for a superficial femoral artery pseudoaneurysm^9^; however, percutaneous drainage in the presence of stent exposure, as observed in the present case, is likely to cause infection, and surgical removal of the hematoma should be considered.

With removal of the infected device, it was necessary to perform bypass of the lower extremity arteries. Generally, the thigh or the medial side of the lower leg is selected as the bypass route in the lower limbs. In case of infection, creation of a bypass route that is as far from the lesion as possible, followed by revascularization, is very important to prevent graft infection. Obturator bypass or lateral femoral bypass is generally used for infections in the common femoral artery region,^10^ and there are few reports of lateral bypass in the superficial femoral artery region. The internal root of the femoropopliteal artery was close to the infected site in the present case; accordingly, the lateral side of the artery was selected as the bypass route. By passing the bypass graft to the lateral side of the femoropopliteal artery, the bypass graft and the wound could be separated from the infected lesion, and revascularization could be performed without causing reinfection.

## 4 CONCLUSION

In the present case, removal of the infected Viabahn with superficial femoral artery–anterior tibial artery bypass surgery via the lateral femoropopliteal route allowed preservation of the lower extremity and prevented graft infection. The findings from this case suggest that although device removal is necessary for treatment, subsequent treatment strategies must be devised to avoid reinfection and preserve the affected extremity.

## AUTHOR CONTRIBUTIONS

**Saeki Watanabe:** Conceptualization; formal analysis; investigation; writing – original draft. **Hironobu Morimoto:** Conceptualization; writing – review and editing. **Daisuke Futagami:** Conceptualization; visualization. **Junya Kitaura:** Conceptualization; visualization. **Shogo Mukai:** Formal analysis; writing – review and editing. **Taira Kobayashi:** Conceptualization; visualization.

## FUNDING INFORMATION

This study did not receive funding from any external sources.

## CONFLICT OF INTEREST STATEMENT

The authors have no conflicts of interest to declare.

## ETHICS STATEMENT

Ethics approval was waived by the local institutional ethics review board.

## CONSENT

Written informed consent was obtained from the patient to publish this report in accordance with the journal's patient consent policy.

## PERMISSION TO REPRODUCE MATERIAL FROM OTHER SOURCES

None.

## CLINICAL TRIAL REGISTRATION

None.

## Data Availability

None.
